# The Relation of Mastication to Physical Development

**Published:** 1890-10

**Authors:** 


### The Relation of Mastication to Physical Development.

Every thing which influences the health of the people is of interest to physicians, and no question more important than dietetics could engage the attention of such a representative body as the American Medical Association. No one could understand the powerful influence which improper food is capable of exerting upon physical development so well as they, and this subject deserves the especial study of American physicians who are desirous of seeing a healthy and vigorous race grow up in this Western Hemisphere. De Toqueville said that the white race in this continent is doomed to extinction. If this ever comes true it will be because of the long-continued neglect of some of the simplest rules of physiology.

That there are at the present time a large number of adults with imperfect teeth is a well-known fact. Poor teeth means poor mastication, poor digestion, poor health, and poor physical development. The early loss of teeth among the people of this country is explained by the unscientific habits of feeding generally practiced among young children. Where the infant is brought up on pap and pre-digested foods the function of mastication is not required. As a result of want of use the jaws imperfectly develop; the arch is narrow and the teeth are crowded and irregular. Nature does not reduce the number of teeth, but she attempts to force thirty-two teeth into jaws that have only room for twenty-four, and the quality of the teeth is not up to the standard, so that they readily commence to decay. When the child has grown up it is too late to prevent the mischief. The decay of teeth is more due to insufficient nourishment than to injury or defect of the enamel.

The rational means of preventing the state of affairs just referred to is to commence early, and give the child food that requires mastication. The result will be increased function of the gums, teeth and salivary glands, and of the masticatory muscles, and the full development of the lower part of the face, with a corresponding improvement in the appearance of the man. In the average family the questions of diet are relegated to the cook, whose study seems to be to provide food which is so soft as not to require to be chewed, and is accompanied by large quantities of coffee, or tea, or ice-water, which takes the place of the salivary secretions. The evil effects of this system of feeding can be seen on every hand. The remedy suggests itself.

Mastication is the most important step; by it the food is reduced to a pulp and is thoroughly incorporated with saliva.

The act of chewing also stimulates the flow of the gastric juice, and is necessary to perfect stomach digestion. General health of the body depends upon digestion and assimilation of sufficient food of proper character, but no matter how much a man regulates his diet he can not altogether overcome the evils of his early training in this direction. Just here we are confronted with a danger which strikes at the very life-blood of the nation, and is already sapping its strength.

If the proper care be observed in rearing children and giving them sound, wholesome food requiring the use of their masticatory muscles, there is no reason why a superior race of men might not be developed, just as we raise the fastest horses and the finest cattle in the world. The appeal is made to physicians especially, to see that the glorious birthright of the American citizen is not bartered away for a mess of pottage or other soft food.

By pursuing the plans adopted by the ancient Greeks, we might not only equal their achivements, but even surpass them in physical development and personal beauty.—Wood, *Dietetic Gazette.*

For tan, freckles, sunburn, etc., equal parts of lemon juice and boro glyceride is as good as anything we know.

